# The 7Ps Framework - Development of a Conceptual Framework on Lifelong Learning Skills

**DOI:** 10.1007/s40670-026-02867-5

**Published:** 2026-09-01

**Authors:** Priyadarshini Dattathreya, Daria Ellis, Alana Headley, Morgan McDaniel, Carlista Tavenier-Durand

**Affiliations:** 1 Hackensack Meridian School of Medicine, Nutley, NJ USA; 2 Hampton University, Hampton, VA USA; 3 Ross University School of Medicine, St. Michael, Barbados

**Keywords:** Lifelong learning, Metacognition, Self-regulated learning, Conceptual framework, Medical student, Feedback

## Abstract

Lifelong learning is a core expectation in medical education yet approaches to cultivating and assessing it are often fragmented. The goal of this study was to develop a practical, theory-informed model of lifelong learning that could (a) unify messaging across our academic success programs (b) to guide diverse applications, including online self-assessment inventories, integration into workshops, development of learning resources, individual coaching sessions, and targeted support interventions (c) help students strengthen their lifelong learning skills, enhance study strategies, and support continuous development, and (d) be applicable to all learners, whether engaged in proactive growth or receiving reactive academic support. We used the MEASURE framework to guide the design of the 7Ps Framework (Plan, Prepare, Participate, Process, Practice, Perform, Pause) and a complementary self-reflection inventory. An exploratory factor analysis (EFA) of 26 items (*N* = 191) revealed a seven-factor structure, accounting for 68.32% of variance, with factor loadings ranging from 0.46 to 0.93 and internal consistency (α = 0.56–0.86). A confirmatory factor analysis (CFA) with a larger sample (*N* = 1,255) demonstrated strong model fit (CFI = 0.94, TLI = 0.93, RMSEA = 0.066), standardized loadings of 0.48–0.91, and acceptable reliability (α = 0.52–0.86). The 7Ps Framework and inventory provide a structured, psychometrically sound approach to promoting lifelong learning and reflection. Ongoing work will further refine the inventory items to strengthen its validity.

## Introduction

The Accreditation Council for Graduate Medical Education (ACGME) defines a set of foundational competencies for undergraduate medical education (UME) that guide training and practice. Among these Practice-Based Learning and Improvement (PBLI) and Professionalism are particularly centered on lifelong learning and development.

PBLI explicitly addresses lifelong learning by emphasizing the integration of feedback, critical reflection, self-assessment, and evidence-informed practice to promote academic performance improvement. Professionalism, in contrast, primarily focuses on ethical conduct, integrity, and accountability. However, sub competencies under Professionalism competencies also support lifelong learning by recognizing that effective professional practice depends on maintaining personal well-being and seeking appropriate support when needed, both of which are integral to sustaining effective professional practice. For example, the sub competency stating that learners should “recognize and address personal wellbeing needs that impact professional performance” acknowledges that self-awareness and proactive management of well-being are essential for learning and performance [1].

Developing these competencies requires learners to draw upon diverse cognitive, behavioral, and motivational strategies that support continuous self-improvement and academic success.

Medical schools attempt to foster these habits through curricular initiatives such as reflective practice, feedback, and goal setting, to develop learners that are continuously adapting to academic challenges [2]. A variety of tools exist to measure self-regulated and lifelong learning skills, ranging from self-assessment questionnaires and inventories to observational methods, and micro-analyses [3–6]. Yet these approaches can be fragmented, as it is challenging to find a single instrument that measures all aspects of lifelong learning [7]. Moreover, self-report measures don’t effectively capture the situational influences on strategy use, and students unfamiliar with learning strategies may misinterpret items, reducing validity [8]. As a result, there is a need for a unifying framework that is theoretically grounded, practically applicable to teaching and assessing lifelong learning skills in multi-modal ways.

To address this gap, the Academy for Teaching and Learning (ATL) at Ross University School of Medicine (RUSM) has developed a structured framework to support the cultivation of lifelong learning skills. The ATL is a team of Medical Education Learning Specialists (MELS) that support the student development of lifelong learning skills through academic success programs, courses and sessions that are both proactive (development-oriented) and reactive (deficit-based). Our proactive efforts include offering online resources, workshops and individualized academic coaching sessions on skills required for success in medical school such as time management, information processing, self-testing, test taking etc. to all students. Our reactive efforts include providing academic success courses to all students remediating a failed semester.

To provide structure and consistent messaging across all our programs, we required a conceptual framework on lifelong learning skills that was grounded in theory, while also memorable and practical. Such a framework was necessary because existing instruments and approaches were fragmented and did not fully capture the complexity of medical students’ needs. Without a unifying structure, our proactive and reactive programming risked becoming inconsistent, and difficult to evaluate systematically. We therefore sought a framework that could be applied in a multimodal way, specifically to teach and assess lifelong learning skills, promote meaningful reflection, and to support student progress over time.

## Materials and Methods

We used the MEASURE framework to guide our process [9]. This approach offered a systematic way to use theory in the development of our conceptual framework and self-assessment inventory. Below, we describe the steps followed in each phase.

### Make the Purpose and Rationale Clear

The primary goal was to provide a practical and theory-informed model of lifelong learning that could unify messaging across academic success programs at RUSM. In developing this model, we sought to integrate elements of PBLI, including reflection, use of evidence, and commitment to cultivating lifelong learning along with elements of Professionalism, such as learning from others and recognizing personal well-being needs that may impact professional performance [1].

The secondary goal was to develop a self-assessment inventory that complemented the framework. The purpose of the inventory was to foster critical self-reflection through objective feedback that supports individualized academic coaching [10, 11]. The framework was intended to be both pedagogically grounded and user-friendly, enabling students to monitor and enhance their lifelong learning skills.

We compared several established models of learning and self-regulation such as the Learning and Study Strategies Inventory (LASSI), the Motivated Strategies for Learning Questionnaire (MSLQ), Learning Strategies of University Students (LIST-K) questionnaire [12–14]. We noted that these inventories assessed learning strategies, motivation, self-regulation and related constructs, but they each emphasized different domains. These tools focused on cognitive and motivational strategies but did not sufficiently integrate wellness strategies which was a critical competence for us. We wanted a framework that also included explicit focus on stress management components that are vital to long-term success and burnout prevention in medical training [15].

Because it was essential that the framework be both user-friendly and practically applicable, we chose not to retrofit an existing model. Instead, we identified the need to synthesize a new, integrative framework that would:

- Incorporate the learning theories essential for lifelong learning.
- Use language and structure that are memorable, actionable, and easily translatable into academic coaching.
- Address both proactive learning behaviors (e.g., planning, preparation, self-testing) and protective strategies (e.g., stress management, recovery, and wellness).

By embedding these priorities, we aimed to develop a framework and a tool that is comprehensive yet practical, and flexible enough to integrate into existing academic support programming while maintaining theoretical rigor.

### Establish Empirical Framework

We began by conducting a narrative review of existing literature on academic success in health professions education, with a focus on metacognitive and self-regulatory skills. We examined both theoretical constructs and practical interventions associated with improved learning outcomes. Three theories were selected to guide the framework development [16]. We identified the following theories to support our framework.

### Self-Regulated Learning (SRL)

SRL is the use of metacognitive skills in a cyclical process where a student sets learning goals, monitors their progress, evaluates their performance and adjusts their goals [17]. We used the principles of SRL as a foundation for our framework to highlight the metacognitive strategies before, during and after a learning event. We also incorporated additional skills such as time management to emphasize the significance of planning and goal setting; and test taking strategies due to its impact on performance [18].

### Sociocultural Theory

According to this theory, knowledge is constructed by learners through interactions with others [19]. The feedback that a learner receives through such interactions, especially with those who have a higher level of knowledge, skill, or experiences, is valuable to inform SRL. Therefore, we used the elements of sociocultural theory in our framework to emphasize the importance of feedback through interactions.

### Theory of Preventative Stress Management (TPSM)

TPSM states that response to stressful stimuli can either be positive (eustress that increases motivation) or negative (that causes physical and emotional distress). Prolonged negative responses to stress are associated with burnout. According to TPSM, negative stress responses can be prevented through interventions that are either primary (that reduce stressors and promote eustress) or secondary (that reduce negative stress responses) [20]. We included mind and body wellness in our framework to emphasize promotion of eustress and prevention of burnout.

Each theory informed specific domains of the eventual framework.

### Articulate Theoretical Blueprint

Based on the synthesis of theoretical and empirical insights, we articulated seven distinct domains essential to lifelong learning, each corresponding to a phase in the learning process:

- Plan – Time management and goal setting.
- Prepare – Organizing and priming before a learning activity.
- Participate – Active engagement during a learning experience.
- Process – Meaning-making and integration of new knowledge.
- Practice – Retrieval and self-assessment.
- Performance – Test-taking and knowledge application under pressure.
- Pause – Self-care, and rest.

We termed this the 7Ps Framework. Each component was linked to a verb to prompt actionable behaviors. We adapted the structure from the “Concise Learning” method and refined it based on theory and practical experiences in academic coaching [21].

All seven components aligned with SRL, while Participate aligned with sociocultural theory and Pause aligned with TPSM [17–20].

### Synthesize Content and Scale Development

Using the 7Ps framework as a theoretical blueprint, we developed a reflective self-assessment inventory to help students regularly evaluate their lifelong learning skills. The use of a theoretical blueprint served two primary purposes: (a) to define the content and domain areas that constitute the construct of lifelong learning within our model, and (b) to guide the proportional distribution of items across each of the seven framework components to ensure balanced representation [9]. Our goal was to ensure that each inventory item aligned clearly with one of the seven domains of the 7Ps framework and reflected observable behaviors or practices relevant to student practice. It was also crucial that the practices were phrased in accessible, student-centered language.

We convened an initial development committee, consisting of four MELS from the Academy for Teaching and Learning (ATL). The MELS had combined expertise in educational principles, learning science, and experience in student support; two were physicians with medical knowledge.

Over several structured brainstorming sessions, the committee translated each of the seven framework components into operationalized behaviors. Approximately 40–45 draft items were generated, with roughly 5–7 items per domain, consistent with the principle of proportional item representation.

Each item was written as a first-person statement (e.g., “I tested my understanding of concepts”) to encourage retrospective self-reflection. This phrasing strategy was intentional, as the inventory was designed to be completed after major learning events (e.g., exams), thereby reinforcing reflective practice [22]. All items used a 5-point Likert-style response scale ranging from “Never” to “Always” (1 = Never, 2 = Sometimes, 3 = About half the times, 4 = Most of the time, 5 = Always).

### Use Expert Reviewers

To establish content validity, we expanded our expert panel to include 12 learning specialists (faculty, academic coaches, and instructional designers) with diverse backgrounds in basic and clinical sciences, medical education and student support [9]. Participants received a complete set of draft items (*n* ≈ 43) and were instructed to perform an independent, unforced digital card-sorting exercise. Each specialist was provided with the items, and the seven categories of the framework. Working independently, each specialist placed every item card under the label that best matched the intended framework component. They were also asked to note any items they felt were ambiguous, overlapping, or poorly worded [23].

After all specialists completed the sorting activity, we calculated the proportion of experts who placed each item under its original intended category. Items with < 80% agreement were flagged for review. We revised or removed the flagged items to improve clarity and categorical fit [24]. This iterative process reduced the item pool to 28 refined items.

### Recruit Participants

To verify that students interpreted and categorized items as intended, we conducted a parallel card-sorting activity with a purposive sample of ten medical students. Each student received the revised items in randomized order and the same set of seven category labels with definitions. Instructions mirrored those given to experts: sort each item under the label that best describes the behavior or strategy addressed. We then computed student‐level agreement percentages and compared them against expert sorting. We identified items which had < 80% of students placed under the intended category and updated them with minor wording tweaks to enhance clarity [24]. Applying the recommendations from Hinkin (1998), we reduced the number of items using both exploratory and confirmatory factor analysis [25].

We obtained approval by the RUSM Institutional Review Board to collect student responses. We piloted the finalized 26-item inventory using an online survey platform following each major summative assessment in the preclinical curriculum. Students were invited to complete the inventory as a voluntary reflective post-exam activity [9].

We collected responses from a convenience sample of first year medical students over a two-year period. Consistent with recommendations for instrument development, our sample size was determined to ensure adequate power for exploratory and confirmatory factor analyses, with over 190 participants included in the pilot and 1,255 participants included in the validation study, exceeding commonly accepted guidelines for factor analytic research [25]. We evaluated the suitability of the data for factor analysis using the Kaiser–Meyer–Olkin (KMO) measure of sampling adequacy and Bartlett’s test of sphericity [26].

We conducted an EFA on the 26 items using principal axis factoring with promax rotation, as the factors were expected to correlate. We used several criteria to determine the number of factors to retain, including eigenvalues greater than 1, visual inspection of the scree plot and conceptual interpretability [27, 28].

### Evaluate Validity and Reliability

After incorporating minor modifications to the survey items informed by the pilot study, we administered the final instrument to first- and second-year medical students. Survey completion was required as part of the post-summative examination process. However, for research purposes, only responses from students who provided informed consent for use of their data were included in the analysis. Students were asked to rate each item of the 26-item instrument on a 5-point Likert scale (1 = Never, 5 = Always).

Using the 26-item scale, we further evaluated the validity of the 7-factor structure applying Confirmatory Factor Analysis (CFA) Lavaan for R [29]. The following fit indices were used for model evaluations: chi-square/*df* ratio (*χ²*/*df*), relative fit indices including the comparative fit index (CFI), the Tucker–Lewis index (TLI), the normed fit index (NFI), parsimony-adjusted measures such as root mean square error of approximation (RMSEA), standardized root mean square residual (SRMR), Akaike information criterion (AIC), and Bayesian information criterion (BIC). For chi-square/*df* ratio (*χ²*/*df*), a score of 3 or less indicates a good fit [30]. CFI and TLI values greater than 0.95, RMSEA values less than 0.06, SRMR values less than 0.05, and lower values AIC and BIC indicate a good fit [30, 31]. 

## Results

### Results of Pilot Study

For the pilot study, we collected responses from a convenience sample of *N* = 191 first year medical students over a two-year period. We computed the item-level descriptive statistics (Table 1). The KMO value was 0.85, exceeding the recommended threshold of 0.60, and Bartlett’s test of sphericity was significant, *p* <.001, indicating that the data were appropriate for factor analysis [26–28].

**Table 1:** Descriptive statistics for the 26 items of the 7Ps lifelong learning inventory pilot study

Item	7Ps Domain	Item Stem	*n*	M	SD
1	**Plan**	I established long-term learning goals for myself.	191	3.87	1.07
2		I broke down larger tasks into smaller short-term goals.	190	3.67	1.10
3		I planned to study multiple disciplines within my daily schedule.	191	4.12	0.96
4		I handled all my learning tasks on time.	190	3.38	1.09
5	**Prepare**	I previewed the learning objectives.	191	3.22	1.32
6		I previewed relevant material to identify familiar concepts.	191	3.00	1.34
7		I previewed to identify concepts that were new to me.	191	2.98	1.35
8		I gathered my resources in preparation for the sessions.	191	3.85	1.25
9	**Participate**	I discussed concepts with other students to gauge my understanding of the material.	191	3.22	1.37
10		I sought help from professor(s) to clarify concepts that were difficult for me.	191	2.34	1.37
11		I compared my class notes with my peers.	190	2.38	1.50
12		I engaged with other students to check my retention of the material.	191	3.02	1.46
13	**Process**	I organized details under larger concepts.	189	3.67	1.18
14		I connected concepts across disciplines and organ systems in my notes.	188	3.42	1.23
15		I compared and contrasted the concepts in my notes.	188	3.49	1.20
16		I related new concepts to information that I already knew.	188	3.80	0.98
17	**Practice**	I used practice questions to connect concepts.	191	4.12	1.00
18		I tested my understanding of concepts.	189	4.02	1.09
19		I recalled important concepts from memory.	191	3.74	1.07
20	**Perform**	I kept track of the amount of time I spent on each question.	191	3.31	1.37
21		I read each question stem and lead-in question carefully before selecting an answer.	191	4.17	0.90
22		I eliminated answer choices that were incorrect.^a^	—	—	—
23	**Pause**	I got optimal hours of sleep each night.	191	3.34	1.26
24		I incorporated some form of physical activity.	190	2.94	1.43
25		I made time to de-stress to avoid burnout.	191	3.22	1.28
26		I took regular breaks.	191	3.44	1.12

Development sample of Doctor of Medicine students. Items were rated on a 5-point Likert-type scale ranging from 1 (*never*) to 5 (*always*). Item numbers correspond to the a priori 7Ps domain assignment established during the blueprinting phase and are used consistently across Tables 1, 2, 3, 4 and 5. Sample sizes vary across items (188 ≤ *n* ≤ 191) because of item-level nonresponse; pairwise deletion was used

^a^Descriptive statistics for Item 22 were not reported for the development sample in the original analysis output and are pending recomputation; values for this item are available for the validation sample (see Table 5)

**Table 2:** Factor loadings and communalities for the promax-rotated seven-factor solution of the 26-item 7Ps lifelong learning inventory

			Rotated Factor Loading							
Item	APriori Domain	Item Stem	1 Process	2 Pause	3 Participate	4 Prepare	5 Practice	6 Plan	7 Perform	Communality
13	Process	I organized details under larger concepts.	**0.76**	−0.06	0.10	0.02	−0.07	0.09	−0.02	0.64
14	Process	I connected concepts across disciplines and organ systems in my notes.	**0.84**	0.02	−0.08	−0.08	0.12	0.01	−0.04	0.72
15	Process	I compared and contrasted the concepts in my notes.	**0.85**	0.08	−0.02	0.01	−0.17	0.11	−0.01	0.73
16	Process	I related new concepts to information that I already knew.	**0.83**	0.13	0.01	−0.09	−0.09	−0.02	0.05	0.67
19	Practice	I recalled important concepts from memory.^ab^	**0.50**	0.00	−0.14	0.08	0.46	−0.04	0.08	0.64
23	Pause	I got optimal hours of sleep each night.	−0.09	**0.66**	−0.09	0.02	0.07	0.12	0.15	0.58
24	Pause	I incorporated some form of physical activity.	−0.06	**0.81**	0.19	0.09	−0.12	0.00	0.03	0.70
25	Pause	I made time to de-stress to avoid burnout.	0.14	**0.89**	0.02	−0.07	−0.02	0.00	−0.07	0.84
26	Pause	I took regular breaks.	0.12	**0.77**	−0.05	−0.01	0.11	−0.04	0.02	0.70
9	Participate	I discussed concepts with other students to gauge my understanding of the material.	0.04	0.01	**0.90**	−0.01	0.00	0.01	0.07	0.85
11	Participate	I compared my class notes with my peers.	0.02	−0.02	**0.90**	−0.01	−0.07	0.02	0.04	0.80
12	Participate	I engaged with other students to check my retention of the material.	−0.07	0.10	**0.89**	−0.02	0.12	0.00	0.03	0.84
5	Prepare	I previewed the learning objectives.	−0.24	0.02	−0.16	**0.93**	0.02	0.15	0.02	0.74
6	Prepare	I previewed relevant material to identify familiar concepts.	0.24	−0.02	0.09	**0.77**	0.09	−0.19	−0.02	0.83
7	Prepare	I previewed to identify concepts that were new to me.	0.09	0.01	0.10	**0.82**	0.08	−0.12	−0.08	0.80
17	Practice	I used practice questions to connect concepts.	−0.15	0.00	0.13	0.06	**0.88**	0.02	−0.03	0.77
18	Practice	I tested my understanding of concepts.	0.00	0.02	−0.07	0.06	**0.91**	0.03	0.05	0.86
10	Participate	I sought help from professor(s) to clarify concepts that were difficult for me.^a^	−0.07	−0.09	0.37	−0.06	0.10	**0.57**	−0.14	0.50
1	Plan	I established long-term learning goals for myself.^b^	0.33	−0.17	−0.02	0.10	−0.06	**0.54**	0.09	0.54
2	Plan	I broke down larger tasks into smaller short-term goals.^b^	**0.37**	−0.14	0.02	−0.05	0.23	**0.32**	0.23	0.57
3	Plan	I planned to study multiple disciplines within my daily schedule.	0.06	0.21	−0.14	−0.11	0.03	**0.72**	−0.05	0.62
4	Plan	I handled all my learning tasks on time.	0.24	0.16	0.08	0.03	0.19	**0.44**	−0.24	0.60
8	Prepare	I gathered my resources in preparation for the sessions.^ab^	0.31	−0.10	0.09	0.19	−0.19	**0.34**	0.05	0.38
20	Perform	I kept track of the amount of time I spent on each question.	0.26	0.07	0.04	0.07	−0.15	−0.20	**0.64**	0.50
21	Perform	I read each question stem and lead-in question carefully before selecting an answer.	−0.26	0.14	−0.04	0.17	−0.11	0.35	**0.60**	0.63
22	Perform	I eliminated answer choices that were incorrect.	−0.01	−0.04	0.11	−0.21	0.22	−0.10	**0.81**	0.71

*N* = 191. Extraction method: principal component analysis. Rotation method: promax with Kaiser normalization. Factors are numbered in order of extraction and labeled with the 7Ps domain each factor represents. Boldface indicates the salient loading(s) defining each item’s factor placement (|λ| ≥ 0.32). Communality values represent the proportion of item variance accounted for by the seven-factor solution. Items are grouped by the factor on which they loaded most strongly

^a^Item loaded most strongly on a factor other than its a priori 7Ps domain. ^b^ Item exhibited a salient secondary loading (|λ| ≥ 0.30) on an additional factor

**Table 3:** Eigenvalues, percentages of variance and cumulative percentages for factors for 26 items

Factor	Eigenvalue	% of variance	Cumulative %
1 Process	8.20	31.53	31.53
2 Pause	2.54	9.78	41.30
3 Participate	1.83	7.03	48.33
4 Prepare	1.64	6.31	54.64
5 Practice	1.34	5.16	59.81
6 Plan	1.24	4.78	64.58
7 Perform	0.97	3.74	68.32

**Table 4:** correlations among extracted factors after promax rotation

Factor	1	2	3	4	5	6	7
(1) Plan	**-0.74**						
(2) Prepare	0.34	**-0.84**					
(3) Participate	0.39	0.13	**-0.82**				
(4) Process	0.47	0.15	0.36	**-0.86**			
(5) Practice	0.46	0.27	0.3	0.19	**-0.83**		
(6) Perform	0.2	0.28	0.09	0.09	0.13	**-0.56**	
(7) Pause	0.41	0.32	0.26	0.32	0.41	0.29	**-0.81**

N-191, Extraction Method: Principal Component Analysis. Rotation Method: Promax with Kaiser Normalization.

Values in parentheses are Cronbach alphas

Internal consistency for Perform (α = 0.56) falls below the conventional 0.70 threshold and should be interpreted as provisional. This value is attributable in part to the small number of items (k= 3) and to the heterogeneity of the test-taking behaviors sampled. Perform scores are therefore reported for descriptive and formative purposes only and are not recommended for high-stakes or individual decision making until the domain is expanded and re-evaluated (see Limitations and Future Research).

**Table 5:** Descriptive statistics for the 26 item 7Ps inventory validity and reliability measurement study

Item	7Ps Domain	Item Stem	*n*	M	SD
1	**Plan**	I established long-term learning goals for myself.	1255	4	1
2		I broke down larger tasks into smaller short-term goals.	1255	3.9	1
3		I planned to study multiple disciplines within my daily schedule.	1255	4.1	1
4		I handled all my learning tasks on time.	1255	3.4	1
5	**Prepare**	I previewed the learning objectives.	1255	3.4	1.3
6		I previewed relevant material to identify familiar concepts.	1255	3.3	1.3
7		I previewed to identify concepts that were new to me.	1255	3.3	1.3
8		I gathered my resources in preparation for the sessions.	1255	3.9	1.2
9	**Participate**	I discussed concepts with other students to gauge my understanding of the material.	1255	3.2	1.3
10		I sought help from professor(s) to clarify concepts that were difficult for me.	1255	2.4	1.4
11		I compared my class notes with my peers.	1255	2.3	1.4
12		I engaged with other students to check my retention of the material.	1255	3	1.4
13	**Process**	I organized details under larger concepts.	1255	3.7	1.1
14		I connected concepts across disciplines and organ systems in my notes.	1255	3.6	1.2
15		I compared and contrasted the concepts in my notes.	1255	3.6	1.2
16		I related new concepts to information that I already knew.	1255	4	1
17	**Practice**	I used practice questions to connect concepts.	1255	4.3	0.9
18		I tested my understanding of concepts.	1255	4.2	1
19		I recalled important concepts from memory.	1255	4	1
20	**Perform**	I kept track of the amount of time I spent on each question.	1255	3.4	1.4
21		I read each question stem and lead-in question carefully before selecting an answer.	1255	4.2	0.9
22		I eliminated answer choices that were incorrect.	1255	4.3	0.9
23	**Pause**	I got optimal hours of sleep each night.	1255	3.5	1.2
24		I incorporated some form of physical activity.	1255	3.2	1.3
25		I made time to de-stress to avoid burnout.	1255	3.3	1.2
26		I took regular breaks.	1255	3.6	1.1

**Fig. 1:**
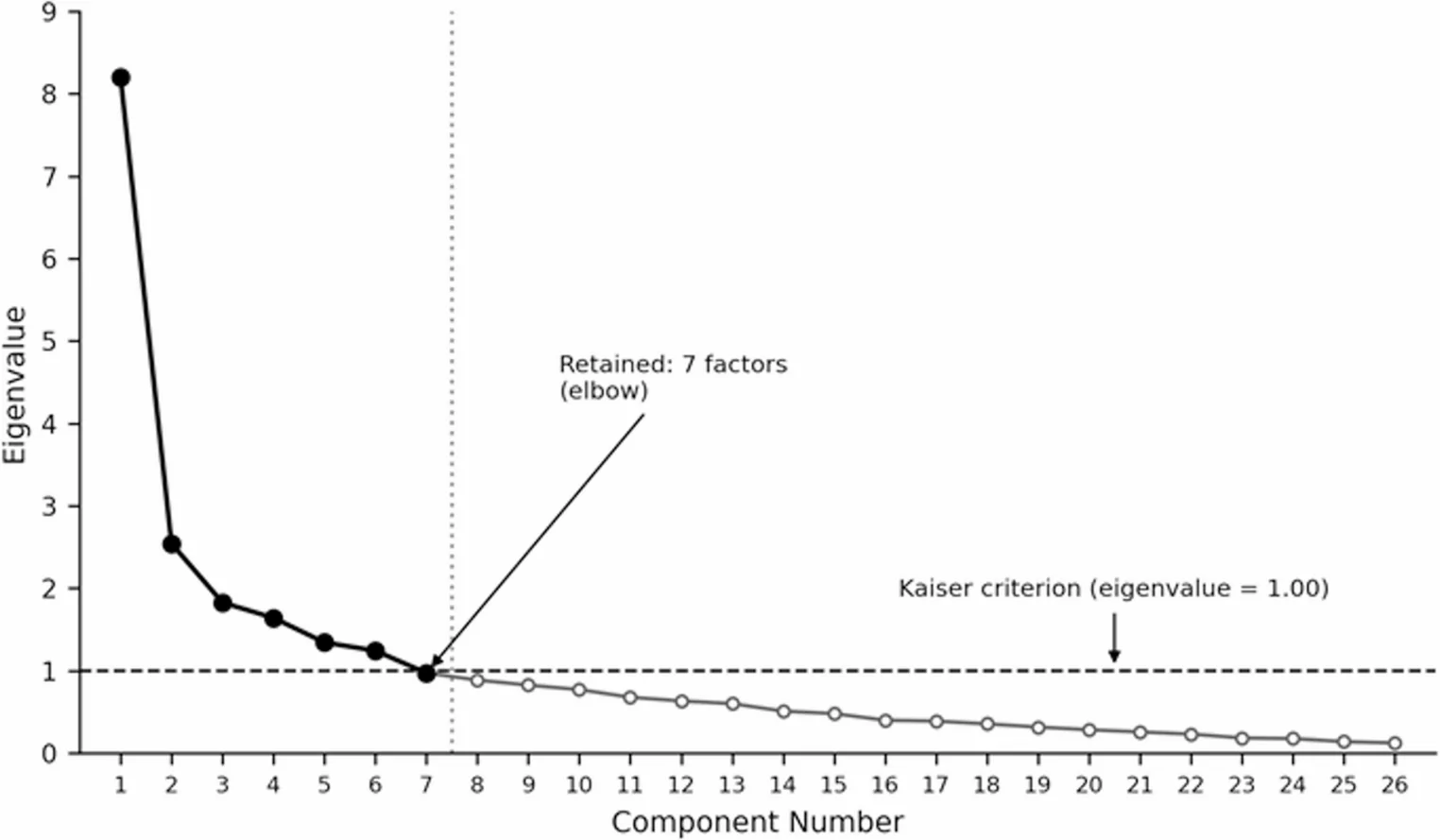
Scree plot of the factor structure for the 26 items. N = 191. The plot displays the eigenvalues of all 26 components extracted from the unrotated principal component analysis solution, ordered from largest to smallest. The horizontal dashed line marks the Kaiser criterion (eigenvalue = 1.00). Filled circles denote the seven components retained for rotation; open circles denote components that were not retained. The curve levels off after the seventh component, and the six components with eigenvalues above 1.00 together with the seventh component at the elbow (eigenvalue = 0.97) jointly accounted for 68.32% of the total item variance. The seventh component was retained because it coincided with the elbow of the curve, was specified a priori in the 7Ps blueprint, and captured a distinct cluster of test-taking behaviors (Perform). Retaining an eighth component would have added only 3.41% of additional explained variance and did not yield an interpretable factor

The EFA supported a seven-factor solution that was both interpretable and parsimonious, with all 26 items retained across the seven factors (see Appendices for details on the EFA analyses and results). The seven factors were labeled *Plan*, *Prepare*, *Participate*, *Process*, *Practice*, *Perform and Pause.* The EFA using principal component analysis with Promax rotation was conducted on 26 items to examine the underlying structure of students’ learning strategies and behaviors (Table 2). The analysis yielded a seven-factor solution, which accounted for 68.32% of the cumulative variance. The eigenvalues for the seven factors ranged from 8.2 (Factor 1) to 0.98 (Factor 7), with each factor contributing between 3.7% and 31.53% of the variance (Table 3). The scree plot further supported the retention of seven factors, as the slope of the curve leveled off after the seventh factor (Fig. 1). Further, most items had communalities ≥ 0.50, indicating good factor representation.

Internal reliability of the 7 factors ranged from marginal to excellent, with Cronbach’s alpha α ranging from 0.56 to 0.86. The factors demonstrated moderate to weak intercorrelations, ranging from 0.11 to 0.60. The strongest correlation was between Factor 1 (Practice) and Factor 2 (Process) (*r* =.60), suggesting that active practice and cognitive processing strategies are closely related (Table 4).

### Results of Validity and Reliability Measurement

We collected survey responses from *N* = 1,255 first- and second-year medical students to evaluate the validity and reliability of the survey instrument.

Descriptive statistics (Table 5) revealed that the highest-rated items were included: “*I eliminated answer choices that were incorrect”* (M = 4.32, SD = 0.90), *“I used practice questions to connect concepts”* (M = 4.31, SD = 0.93), and “*I read each question stem and lead-in question carefully before selecting an answer”* (M = 4.23, SD = 0.90). Whereas the lowest-rated items were “*I sought help from professor(s) to clarify difficult concepts”* (M = 2.39, SD = 1.35) and “*I compared my class notes with my peers”* (M = 2.33, SD = 1.40).

Results of the CFA indicated that the model is a good fit based on commonly accepted thresholds: χ2 = 509.32, df = 278, *p* <.01, RMSEA = 0.066), CFI and TLI values were 0.94, 0.93, respectively. Further, internal reliability of the 7 factors ranged from marginal to excellent, with Cronbach’s α ranging from 0.52 to 0.86.

## Discussion

Developing conceptual frameworks is an iterative process. The underlying reasonings, and the judgements involved in the process are often underreported in literature. Applying the MEASURE to develop the 7Ps framework and the inventory addressed this by providing a sequence of explicit and documentable steps [9]. There were several distinct advantages to using this systematic approach.

First, the MEASURE framework provided us with intentionality. Each phase required us to articulate our objectives before proceeding to the next one. Second, it fostered evidence-based decision making. Each step outlined in the framework was empirically supported and required to be substantiated by existing literature and/or predefined benchmarks [9]. For instance, during the establishment of our empirical framework we explicitly outlined the competencies for inclusion in the framework and identified those that were beyond our immediate scope. As our priority was academic success skills, we distinguished between sub competencies under Professionalism for UME such as managing wellbeing needs with those like compassion and respect that are less directly correlated with academic performance [1, 2]. Third, it structured collaborations by outlining clearly defined points where the process needed group consensus. For example, there were items in the first draft that had around 80% consensus among the expert reviewers but were scored < 70% by the students. This was considered as not strong consensus, so the item was removed. This data-driven decision avoided individual or group biases [9].

While the process of developing the 7Ps framework remained iterative and non-linear, the MEASURE framework provided us with the scaffolding necessary to maintain strategic direction while also allowing for flexibility. Our findings extended previous research by offering an updated framework for lifelong learning skills in medical education. Prior tools, such as the Learning and Study Strategies Inventory (LASSI) and the Motivated Strategies for Learning Questionnaire (MSLQ), emphasized discrete domains like motivation or metacognition but overlooked resilience and stress management [3, 6]. By integrating self-regulated learning, sociocultural theory, and preventative stress management, the 7Ps framework provides a more comprehensive model. The seven distinct yet interrelated factors confirmed in our analyses align with, but also advance, earlier fragmented approaches by simplifying complex constructs into memorable, actionable strategies that students can apply across contexts. Additionally, the framework simplifies the multiple dimensions of lifelong learning for students without the use of jargon, using the 7Ps: Plan, Prepare, Participate, Process, Practice, Performance, and Pause. These verbs capture the regulation of cognition in a way that is accessible and memorable. While this simplification may overstate the clarity of what are often complex and overlapping processes, it offers students an entry point for metacognitive reflection and application that is otherwise difficult to achieve in the medical curriculum [11].

Although the overall factor structure aligned with the proposed framework, several items showed statistical discrepancies. Specifically, a subset of items demonstrated weak factor loadings (< 0.50) or problematic cross-loadings, which may obscure the distinctiveness of the intended constructs [26–28]. For instance, the item “I handled all my learning tasks on time” loaded weakly under the PLAN domain. Similarly, “I broke down larger tasks into smaller short-term goals” showed insufficient loading on its primary factor, PLAN, while simultaneously cross-loading onto PROCESS. Furthermore, the item “I gathered my resources in preparation for the sessions” demonstrated ambiguity by loading across both PREPARE and PLAN domains. These discrepancies may reflect potential ambiguity in wording of these items and may reduce the distinctiveness of the constructs being measured [27]. Consequently, these items require further review to determine whether they should be revised or removed.

Additionally, we recognize that the inventory items themselves are intentionally generic, designed to capture broad patterns of strategy use rather than context-specific behaviors. As such, the greatest value emerges when the inventory is paired with workshops, online materials, and guided coaching conversations with MELS. These contexts allow students to unpack their responses, situate them within the demands of a particular course or assessment, and explore concrete adjustments to their approach. While the inventory encourages the initial reflection and self-reporting of strategy use, when used in conjunction with think-aloud protocols and coaching, it provides the necessary scaffolding to move from awareness to meaningful change [7]. Administering the inventory sequentially after each examination allows longitudinal patterns to offer deeper insights into how persistently each strategy is used. Furthermore, a Likert scale spanning from Never to Always can measure the frequency of implementation for these strategies.

### Limitations

Several limitations should be considered when interpreting our findings. First, the current inventory may lack comprehensive coverage, potentially omitting several critical strategies. This gap combined with weaker performing items noted above, points to a clear need for further refining of the instrument.

Second, the study was conducted within a single institutional context, involving a relatively homogenous group of learners despite some diversity in the student population. This limits the generalizability of the results to other institutions seeking to implement our framework. Thirdly, the use of self-reported survey data introduces potential biases, including recall bias and social desirability bias, as students may have misestimated their engagement with self-directed learning practices [32]. And finally, although the survey was administered multiple times following various assessments, the cross-sectional nature of the data collection limits our ability to draw conclusions about the evolution of study behaviors over time or their impact on long-term academic outcomes [33].

### Future Research

Our future research will aim to include input from a broader group of educators including pre-clinical and clinical content experts to further refine the instrument. Longitudinal studies are needed to explore how self-directed learning strategies evolve throughout medical education and how they relate to standardized assessments and clinical reasoning [33]. Additionally, interventional research could examine the integration of the framework into curricula, mentorship programs, and technology-enhanced learning environments. These studies would help determine the most effective ways to implement self-directed learning principles to improve academic performance and support the development of long-term learning skills essential for medical practice.

## Conclusion

We developed the 7Ps framework and inventory to provide a pedagogically grounded and user-friendly model to support lifelong learning skill development of our medical students. The use of the MEASURE approach provided us with a structured, evidence-based, and systematic approach to develop a framework that includes key competencies required for academic performance and is grounded in theory. We aim to continue refining our inventory and collect data on its use among students and MELS.

## Declarations

### Data Availability

The data that support the findings of this study are available from the corresponding author upon reasonable request.
